# Identification and Characterization of Potato Zebra Chip Resistance Among Wild *Solanum* Species

**DOI:** 10.3389/fmicb.2022.857493

**Published:** 2022-07-27

**Authors:** Victoria Mora, Manikandan Ramasamy, Mona B. Damaj, Sonia Irigoyen, Veronica Ancona, Carlos A. Avila, Maria Isabel Vales, Freddy Ibanez, Kranthi K. Mandadi

**Affiliations:** ^1^Texas A&M AgriLife Research and Extension Center, Weslaco, TX, United States; ^2^Department of Agriculture, Agribusiness, and Environmental Sciences, Texas A&M University-Kingsville, Weslaco, TX, United States; ^3^Department of Horticultural Sciences, Texas A&M University, College Station, TX, United States; ^4^Department of Entomology, Texas A&M University, College Station, TX, United States; ^5^Department of Plant Pathology & Microbiology, Texas A&M University, College Station, TX, United States; ^6^Institute for Advancing Health Through Agriculture, Texas A&M AgriLife, College Station, TX, United States

**Keywords:** *Candidatus* Liberibacter solanacearum, Fastidious bacteria, *Bactericera cockerelli*, zebra chip (ZC), wild accessions, resistant traits, antibiosis

## Abstract

Potato zebra chip (ZC) disease, associated with the uncultured phloem-limited bacterium, *Candidatus* Liberibacter solanacearum (*C*Lso), is transmitted by the potato psyllid *Bactericera cockerelli*. Potato ZC disease poses a significant threat to potato production worldwide. Current management practices mainly rely on the control of the psyllid to limit the spread of *C*Lso. The present study investigated new sources of ZC resistance among wild *Solanum* species. A taxonomically diverse collection of tuber-bearing *Solanum* species was screened; one ZC-resistant accession and three ZC-tolerant accessions were identified among the 52 screened accessions. Further characterization of the resistant accession showed that the resistance was primarily associated with antibiosis effects due to differences in leaf trichome density and morphology of the wild accession, which could limit the psyllid feeding and oviposition. This germplasm offers a good resource for further understanding ZC and psyllid resistance mechanisms, contributing to potato breeding efforts to develop ZC resistance cultivars. Alternatively, it could be used as a potential trap crop to manage psyllid and control ZC disease.

## Introduction

Potato (*Solanum tuberosum* L.) is cultivated in over 160 countries and is rated as the fourth most important staple food crop after wheat, corn, and rice. It is a rich source of carbohydrates and provides other essential nutrients, such as dietary fiber, vitamins, minerals, protein, and antioxidants (Dahal et al., 2019). Based on its global consumption, nutritional benefits, tonnage production, and cash value, the global production was estimated at ~395 metric tons in 2019. Its importance in the United States was at 3.94 billion (Faostat, 2020; USDA, 2020). However, increasing the incidence of biotic (diseases and pests) as well as abiotic (drought, heat, and salinity) stresses limit potato production (Savary et al., 2019).

Since its identification in 1994 in Mexico and in 2000 in the United States, the zebra chip (ZC) disease of potato has spread into several commercial potato-growing regions of Central America, Australia, New Zealand, Northern Africa, and the Middle East (Munyaneza et al., 2007, 2009a, 2010; Tahzima et al., 2014; Mawassi et al., 2018; Mora et al., 2021) and recently in Spain (Portal, 2017). ZC disease could result in potato yield losses of up to 94% (Greenway, 2014). The disease is associated with the uncultured phloem-limited bacterium *Candidatus* Liberibacter solanacearum (*C*Lso) and vectored by the potato psyllid *Bactericera cockerelli* Šulc (Hemiptera: Triozidae) (Munyaneza, 2012; Nwugo et al., 2017; Mora et al., 2021). Typical foliar symptoms of ZC-affected plants include purplish discoloration/chlorosis of young leaves, upward rolling of top leaves, presence of axillary buds, presence of aerial tubers, wilting, stunted growth, and ultimately plant death. ZC symptoms in tubers are associated with the synthesis and accumulation of phenolic compounds, reducing sugars and defense enzymes (Navarre et al., 2009; Wallis et al., 2012), giving chips a bitter taste and a dark brown striped, zebra-like appearance when fried (Munyaneza, 2012), ultimately causing the entire tubers to become unmarketable (Mora et al., 2021). If left uncontrolled, ZC disease can become one of the potato's economically significant diseases.

Current ZC management strategies rely on controlling the psyllid vector, involving insecticides (Guenthner et al., 2012; Greenway, 2014; Greenway and Rondon, 2018) and the possibility of increased insecticide resistance. Recently, resistance to neonicotinoid-based insecticides has been reported in Texas potato psyllids, which threatens future control of this insect (Prager et al., 2013; Szczepaniec et al., 2019). The primary mode of acquisition and spreading of *C*Lso is by psyllids feeding on *C*Lso-infected plants and then on healthy plants transferring *C*Lso (Munyaneza et al., 2009b; Buchman et al., 2011). Hence, identifying novel genetic resistance and tolerance to *C*Lso or the psyllid can be valuable components of ZC's integrated pest/diseases management. Previous studies have reported variations in the psyllid preference for the wild potato species, namely, *Solanum bulbocastanum, Solanum habrochaites*, and *Solanum Verrucosum*, and the breeding clones, namely, *Solanum berthaultii* and *Solanum tuberosum* (Butler et al., 2011; Cooper and Bamberg, 2014, 2016; Diaz-Montano et al., 2014; Levy and Tamborindeguy, 2014). Variations in the tolerance response to *C*Lso have also been noted among various *Solanum* breeding clones (Prager et al., 2013; Rashidi et al., 2017; Vigue, 2018; Fife et al., 2020; Vigue et al., 2020).

A 2015 taxonomic and genetic study established a *Solanum* section *Petota* panel of tuber-bearing relatives of cultivated potatoes, representing a genetic resource of broadly diverse germplasm of highly regarded agronomic traits and tuber size (Hawkes, 1990; Spooner and Castillo, 1997; Hardigan et al., 2015). In this study, screening and identification of new ZC-resistant and -tolerant germplasm in the *Solanum* sect. *Petota* panels were performed. The ZC resistance of one accession (*S. berthaultii*) could be attributed to modifications in leaf trichome shape and density, affecting potato psyllid fecundity and survival.

## Materials and Methods

### Plant Materials, Propagation, and Maintenance

Plant material consisted of 52 wild potato accessions grown from true potato seeds obtained from the U.S. National Plant Germplasm System (NPGS) in Wisconsin, USA. The introductions belong to the *Solanum* sect. *Petota* diversity panel (Supplementary Table 1). This panel represents the germplasm of tuber-bearing *Solanum* species exhibiting morphological variations (Supplementary Figure 1) (Hardigan et al., 2015). *Solanum tuberosum* L. var. Atlantic (chip processing market class) was used as a susceptible control in all experiments and was initially propagated from certified disease-free seed tubers. For *in vitro* germination and propagation, the *Solanum* botanical seeds were pretreated with 10% (w/v) gibberellic acid (GA3) overnight in a 1.5 ml microcentrifuge tube to break dormancy and enhance seed germination. Furthermore, seeds were surface sterilized in 70% (v/v) ethanol for 3 min and then with 10% (v/v) bleach and 2% (v/v) Tween^®^ 20 (Sigma-Aldrich, St. Louis, MO, USA) for 10 min, followed by rinsing four times with sterile water. Sterilized seeds were placed on a sterile wetted Whatman™ paper inside a sterile 100 × 25 mm Petri dish and kept at 22°C in the dark for 1 week. Germinated seeds were grown on Murashige and Skoog (Murashige and Skoog, 1962) solid media supplemented with pre-made MS vitamins (Caisson Labs, North Logan, Utah, USA), 2% sucrose (w/v), and 2 g/L of Gelrite™ (Research Products International, Mt. Prospect, IL, USA), pH 5.8. When plants were 4 weeks old (about 8 cm tall), micropropagation was done using internode cutting as explants; sub-culturing every 4 weeks was necessary to increase plant material for each wild potato accession for screening against ZC. All explants were maintained in a temperature-controlled growth chamber at 22°C under 14-h light/10-h dark photoperiod.

### Psyllid Maintenance

Potato psyllid (*Bactericera cockerelli* Šulc.) colonies consisted of *C*Lso-free (*C*Lso^−^) and *C*Lso-positive (*C*Lso^+^) haplotype B, which were obtained and reared for several generations at Texas A&M AgriLife Research and Extension Center in Weslaco in 60 × 60 × 60 cm nylon mesh cages (Bugdorm, BioQuip Products Rancho Dominguez, CA, USA). Psyllid colonies were reared and maintained on potato plants (Atlantic) and periodically diagnosed for the presence of *C*Lso (>90% *C*Lso^+^) and the absence of *C*Lso (*C*Lso^−^) by polymerase chain reaction (PCR) using genomic DNA isolated from adult psyllids (Sengoda et al., 2014) and primers specific to the 16s rDNA of *C*Lso (OA2-F and OI2c-R) (Supplementary Table 2) (French-Monar et al., 2010). PCR conditions were given as follows: one denaturing cycle at 95°C for 30 s; 35 cycles each at 95°C for 30 s, 68°C for 30 s, and 68°C for 2 min; and a final extension cycle at 68°C for 5 min. PCR amplicons were separated by electrophoresis on a 1.0% (w/v) agarose gel stained with ethidium bromide (0.5 μg/ml).

### Screening of Wild *Solanum* Sect. *Petota* Accessions for ZC Resistance

For primary screening, 52 wild potato accessions were grown in a professional growth mix (Berger BM6 All-Purpose mix, pH 5.4–6.2) in 2.84-l pots at 22°C under 14-h light/10-h dark photoperiod and 50% relative humidity in controlled growth chambers. No choice feeding was performed on 4-week-old plants by inoculating each plant with 20 *C*Lso^+^ psyllids enclosed into two separate organza drawstring bags (10 psyllids/bag), which were tied to the second and third fully expanded leaves (Supplementary Figure 2B), with four replicates of inoculated plants per accession. The bags and psyllids were removed from plants 1 week after placement. Phenotypic evaluations of foliar ZC disease symptoms were done at 14, 21, and 28 days post-inoculation (dpis) in an environment-controlled growth chamber. Foliar symptoms were evaluated and rated based on a scale of 1–3, where 1 = being resistant (no symptoms), 2 = moderately susceptible (some symptoms), and 3 = highly susceptible (severe symptoms and morbidity) (Supplementary Figures 2C, 3). Chip frying and ZC scoring were performed as described in other studies (Henne et al., 2010; Harrison et al., 2019).

A second screening of the ZC resistant and moderately susceptible accessions chosen from the primary screening and a screening of the single seed lines from true potato seeds were done using 1-month-old plants. To increase quantity, all plants were vegetatively propagated by tissue culture and were planted in modified enclosed transparent 32 oz. plastic cups. No choice feeding was performed by releasing five *C*Lso^+^ psyllids (presence of *C*Lso was confirmed by PCR diagnostics) into each enclosed cup containing two plants in a professional growth mix Berger BM6 All-Purpose mix, pH 5.4–6.2, with two cups per accession. *C*Lso^−^ psyllid (absence of *C*Lso was confirmed by PCR diagnostics) challenged and non-challenged plants were used as negative controls (Supplementary Figure 2A). Phenotypic evaluations of inoculated plants consisted of assessing foliar symptoms of ZC at 14, 21, and 28 dpi. At 7 dpi, the soil was drenched with Admire Pro (Bayer Crop Science, Germany) to kill all psyllids in enclosed cups and prevent further plant damage. Leaf tissue was collected at 21 and 28 dpi, with 3–4 leaves from the top, middle, and bottom of each plant, cut into pieces with a single edge blade, and pooled to produce four replicates before freezing in liquid nitrogen (VWR Reinforced 2 ml Bead Mill Tubes, Radnor, PA, USA). Tissues were lyophilized before genomic DNA extraction for PCR and quantitative PCR (qPCR) analyses.

### Molecular Diagnostics for *C*Lso Detection and Quantification

Genomic DNA was extracted from leaf tissue using a modified protocol as previously published (Edwards et al., 1991). For the detection of *C*Lso in tissues collected from wild potato accessions and Atlantic control, conventional PCR was performed on a ProFlex™ PCR System (Applied Biosystems, Life Technologies, Carlsbad, CA) in a total reaction volume of 20 μl, using 150 ng of DNA, 0.5 μM of each target-specific primer, 10 μl of AccuStart Tough Mix (Quantabio, Beverly, MA, USA), 0.5 μl of 50 × loading dye, and 6.6 μl of nuclease-free water (Ambion, Life Technologies, Austin, TX, USA). For initial conventional (gel) PCR analysis, a primer pair, OI2C-F and OA2-R, was used to specifically amplify *C*Lso 16S rDNA (Levy et al., 2011). The potato *Ribosomal Protein L2(RPL2)*-specific primers RPL2-F and RPL2-R were used to amplify an endogenous reference gene (Supplementary Table 2) (Avila et al., 2012). PCR conditions were given as follows: one denaturing cycle at 95°C for 30 s; 28 cycles each at 95°C for 30 s, 50°C for 30 s, and 68°C for 2 min; and a final extension cycle at 68°C for 5 min. PCR amplicons were separated by electrophoresis on a 1.0% (w/v) agarose gel stained with ethidium bromide (0.5 μg/ml).

The *C*Lso relative titer was quantified in tissues collected from wild potato accessions and Atlantic control using quantitative PCR (qPCR) in a CFX384™ Real-Time System (Bio-Rad Laboratories, Inc., Hercules, CA, USA) in a total reaction of 10 μl, using 50 ng of DNA, 0.4 μM of each target-specific primer, and 5 μl of i*Taq*™ Universal SYBR Green Supermix (Bio-Rad Laboratories, Inc., Hercules, CA, USA). Three biological replicates were used with two technical replicates. Lso-F and HLB-R primers (Supplementary Table 2) were used to specifically amplify *C*Lso 16S rDNA, while Sotu-RPL2-F and Sotu-RPL2-R primers (Supplementary Table 2) were used for the amplification of an endogenous reference gene, *RPL2* (Levy et al., 2011; Irigoyen et al., 2020). PCR conditions were given as follows: one denaturing cycle at 95°C for 3 min; 40 two-step cycles each at 95°C for 15 s and at 55°C for 30 s; and a final melting curve of 65–95°C for 55 s. The results were analyzed and recorded as C_T_ (threshold cycle) values, which were normalized against the C_T_ values of the potato *RPL2* (Supplementary Table 2) for quantification using the comparative C_T_ method ($2_{\text{T}}^{-\text{ΔΔ}C}$) (Zuñiga et al., 2020). The Student's *t*-test was used to determine statistically significant (*p* ≤ 0.05 or 0.01) differences between the controls and treatments.

### Host Selection and Olfactometer Assays

The olfactory host-preference assays of *B. cockerelli* adults (*C*Lso^−^), reared and maintained on Atlantic potato plants, were conducted using 2-month-old *Sb-*PI310927 and Atlantic plants and a Y-tube olfactometer (35 cm long × 2.5 cm diameter). Twenty *B. cockerelli* adults per set of plants (*n* = 10 sets of plants, *Sb-*PI310927 and Atlantic plants) were collected into plastic vials for 16 h before initiating the behavioral assays to promote insect host choice. The Y-tube was connected to a 2-port Humidified Air Delivery system (ARS, Gainesville, FL, USA). The air was filtered through an activated carbon filter (16 cm, ARS). Airflow of each arm (bifurcated at a 45 angle) was set up at a constant rate of 10 L/min, leading into two separated sealed cylindrical glass chambers (15 cm diameter, 35 cm high) that contained individual plants, *Sb-*PI310927 or Atlantic, as odor sources. The Y-tube was placed at a 30° angle from the surface and adult psyllids were released at the end of the tube. To examine the insect olfactory behavior, observations were recorded by counting the number of psyllids at each arm's terminal end (set threshold = 4 cm) every 15 min for a 60-min period. All others were considered as a non-responding group. All measurements were conducted between 2 and 4 p.m. Central Standard Time (CST) at ambient temperature (21°C) and under constant light (~250 μmol/m^2^ s).

### Survival and Oviposition of *B. cockerelli* on *Sb-*PI310927 and Atlantic Plants

The survival of *B. cockerelli* adults (*C*Lso^−^) on 2-month-old *Sb-*PI310927 and Atlantic plants (*n* = 10 plants; 10 psyllids/plant) was evaluated to record the surviving insects every day for 7 days. To examine the oviposition of *B. cockerelli* on *Sb-*PI310927 and Atlantic plants, a non-choice assay was performed on insect-proof mesh cages (30 × 30 × 30 cm). Before the assay was initiated, couples (female and male psyllids) were allowed to mate for 4 h in a 1.7-ml tube and subsequently transferred onto plants (*n* = 10 plants; 10 psyllids/plant). After 7 days, adult psyllids were removed, and the total number of eggs was recorded.

### Trichome Evaluation of *Sb-*PI310927

One-month-old *in vitro* culture plants of ZC-resistant single seed line 10 of *Sb-*PI310927 accession and Atlantic control were hardened for 1 month in the growth mix. Three 2-mm diameter disks were excised from one leaflet of the second and third fully expanded leaves from two plants of each of *Sb-*PI310927-resistant line 10 and Atlantic control, using a 2-mm sampling tool (Electron Microscopy Sciences, Hatfield, PA, USA), and observed at 60 × on an Olympus BX51 stereomicroscope (Olympus Corporation, Tokyo, Japan). Leaflet disks were evaluated for leaf trichome shape and density based on two biological samplings and 12 technical replications. Trichome density was calculated as the number of trichomes per mm^2^ leaf area on both the adaxial and abaxial leaflet surfaces.

### Data Analysis and Statistics

The data analysis, statistics, and graphs were displayed as described in the figure legends using the Microsoft Excel software (version 2009). The Student's *t*-test was used to determine statistically significant (*p* ≤ 0.05, 0.01, or 0.001) differences in *C*Lso titer and oviposition between control and treatment. The Chi-square (χ^2^) test was used to compare the number of potato psyllids responding to plant odor sources using a Y-tube olfactometer. The survival of potato psyllids was estimated using the Kaplan–Meier survival analysis using the RStudio environment (Allaire, 2011).

## Results

### ZC Resistance Exists Among the Wild *Solanum* Sect. *Petota* Collection

**Figure 1:**
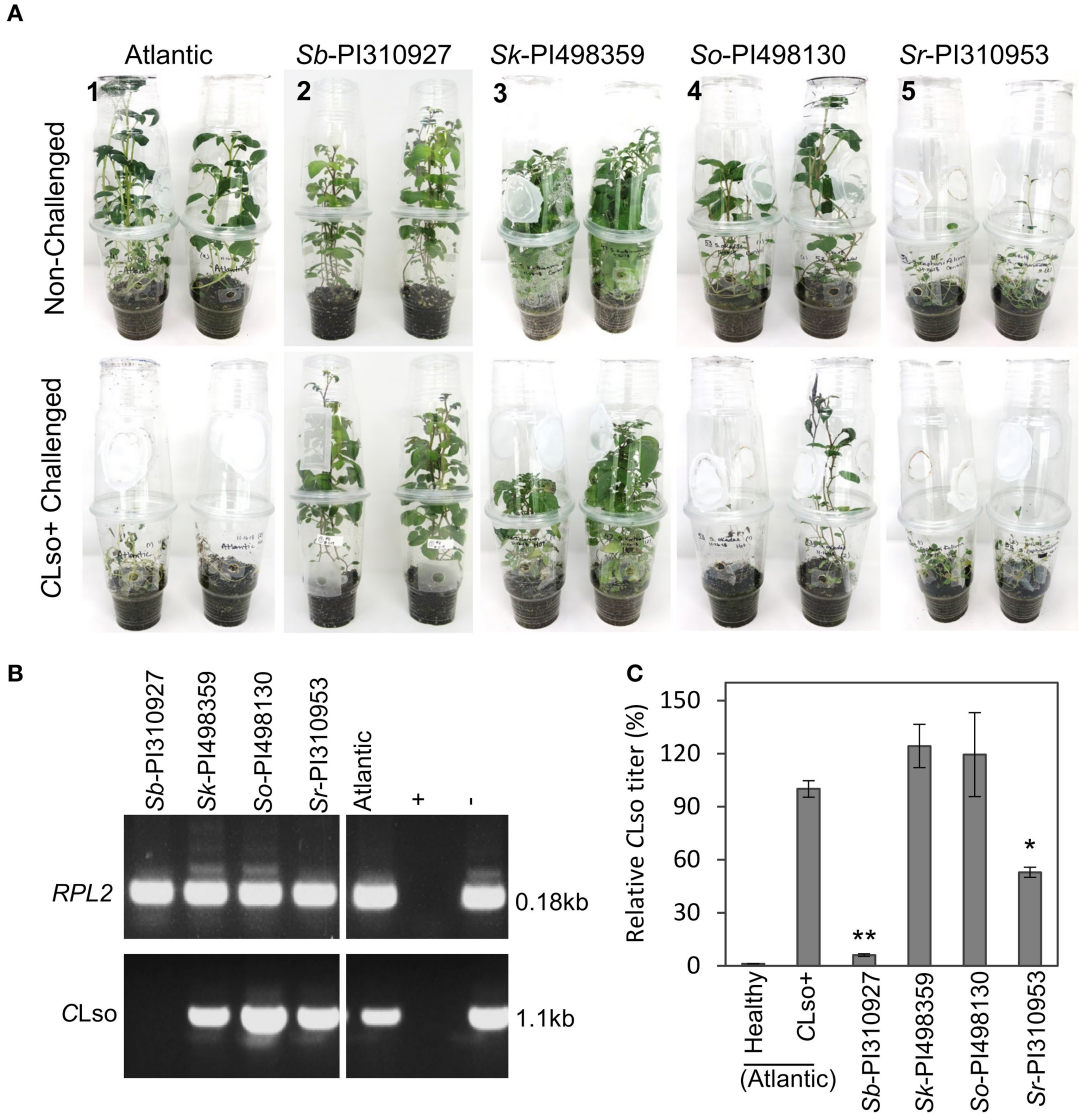
Phenotypic evaluation and *C*Lso quantification of ZC resistant and tolerant wild *Solanum* accessions. A no-choice psyllid feeding assay was performed by releasing five *C*Lso^+^ psyllids on two plants (1-month-old) in a professional growth mix per enclosed cup in an environment-controlled growth chamber. (**A**) The top panel shows representative photos of non-challenged accessions, and the bottom panel representative photos of *C*Lso^+^ challenged accessions at 28 dpi [1, Atlantic control, severity index (SI) = 3. 2; *S. berthaultii PI310927*, SI = 1; 3, *S. kurtzianum* PI498359, SI = 2; 4, *S. okadae* PI498130, SI = 2. 5; and *S. raphanifolium* PI310953, SI = 2). (**B**) Detection of *C*Lso by PCR amplification of *C*Lso 16s rDNA in leaf tissues of the resistant accession (*Sb*-PI310927) and the three tolerant accessions (*Sk*-PI498359, *So*-PI498130, and *Sr*-PI310953) at 28 days post-inoculation (dpi). The potato *RPL2* endogenous gene was used as the PCR control. (**C**) Relative quantification of *C*Lso titers of the accessions at 28 dpi by qPCR. Relative *C*Lso titers were calculated from three biological replicates; error bars represent the mean *C*Lso titer ± standard error of the mean. The *p*-values were calculated by Student's *t*-test relative to the infected control. *, **P ≤ 0.05 and 0.01, respectively.

Primary screening of 52 wild potato accessions was performed from the *Solanum* sect. *Petota* collection (Supplementary Table 1) to identify potential lines with resistance and tolerance to *C*Lso or the psyllid. Multiple *in vitro* propagated plants of each accession were hardened in pots for 2 months, followed by exposure to *C*Lso^+^ psyllids. Foliar symptoms of ZC were then observed every 7 days for up to 4 weeks (Supplemental Figure 2A, B) and rated based on a scale of 1–3, where 1 = being resistant (no symptoms), 2 = moderately susceptible (some symptoms), and 3 = highly susceptible (severe symptoms and morbidity) (Supplementary Figure 2C). Several of the 52 accessions were susceptible and moderately susceptible, showing some upward leaf rolling, chlorosis, and plant stunting, but they grew new leaves at 21–28 dpi. Interestingly, one diploid accession *Solanum berthaultii* Hawkes (*Sb-*PI310927), sourced initially from Bolivia, seemed resistant with no visible ZC symptoms. The nine accessions (one resistant and eight moderately susceptible) were selected, and the challenges with *C*Lso^+^ psyllids were repeated using 1-month-old plants grown in terrariums (enclosed cups). In addition to phenotypic observation for ZC symptoms, the relative *C*Lso titers were estimated at 28 dpi when *C*Lso could be sufficiently detected. The single resistant accession, *Sb-*PI310927, reproducibly showed no visible symptoms and continued to grow after 28 dpi with a severity index of 1, compared with the susceptible Atlantic control that displayed severe symptoms and died at 28 dpi (severity index 3). Three accessions, namely, *Sk*-PI498359, *So*-PI498130, and *Sr*-PI310953, again showed reproducible ZC symptoms with a severity index scale of 2 (Figure 1A). The remaining five accessions displayed variable symptoms and died at 28 dpi, with a severity index of 3. In this experiment, both conventional and quantitative PCR confirmed the presence or absence of *C*Lso (Figures 1B,C). The resistant accession *Sb*-PI310927 and the three moderately susceptible accessions (i.e., *Sk*-PI498359, *So*-PI498130, and *Sr*-PI310953) displayed *C*Lso titers of 6.05% and 52.44–119.36%, respectively, relative to that of the Atlantic control (set to 100%) (Figure 1C). Further comparative studies were carried on with the resistant (*Sb*-PI310927) and highly susceptible control (Atlantic) plants.

### *Sb-PI310927* Is ZC Resistant

**Figure 2:**
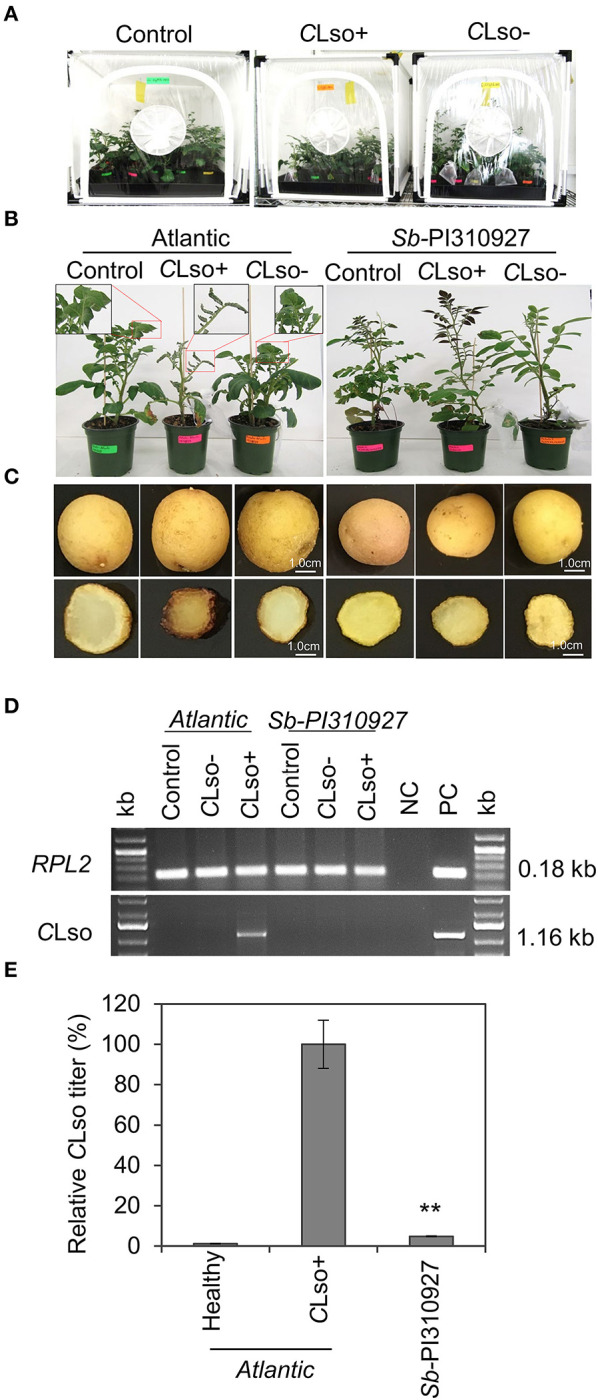
ZC resistance trait evaluation of the *Solanum berthaultii* accession PI310927. (**A**) Two-month-old *Sb*-PI310927 accession and Atlantic control plants in pots were randomized and challenged with *C*Lso^+^ and *C*Lso^−^ psyllids inside cages under greenhouse conditions. (**B, C**) The above-ground phenotype of 4-week-old *Sb*-PI310927 accession and Atlantic plants and their corresponding tuber and fried chip phenotypes. (**D**) Detection of *C*Lso by PCR amplification of *C*Lso 16s rDNA in leaf tissues of *Sb*-PI310927 accession and Atlantic at 28 dpi. The potato *RPL2* endogenous gene was used as the PCR control (NC, negative control; PC, positive control). (**E**) Relative quantification of *C*Lso titer of *Sb*-PI310927 accession and Atlantic control plants at 28 dpi by qPCR. Relative *C*Lso titers were calculated from three biological replicates; error bars represent ± standard error of the mean. The *p*-values were calculated by Student's *t*-test relative to the infected control. **P ≤ 0.01.

The ZC resistance trait of *Sb*-PI310927 was confirmed by repeating the ZC challenges, using 2-month-old plants under greenhouse conditions, and evaluating the ZC tuber symptoms. Typically, *C*Lso-psyllid feeding for the brief period of the challenge (~7 days) causes the inoculated lower leaves to wilt, while the newly emerging (upper) non-inoculated leaves remain asymptomatic. In contrast, *C*Lso+ psyllids induce symptoms associated with ZC on new upper non-inoculated leaves ranging from leaf wilting, upward curling, and chlorosis/necrosis. They can also lead to severe stunting of the plants (Supplementary Figure 4). *Sb*-PI310927 plants consistently showed no visual ZC foliar symptoms with a severity index of 1 when challenged with *C*Lso^+^ or *C*Lso^−^ psyllids. However, the susceptible Atlantic variety displayed typical ZC foliar symptoms with a severity index of 3 when infected with *C*Lso^+^ psyllids at 28 dpi (Figure 2B). Freshly cut tubers and fried chips of *C*Lso-infected *Sb*-PI310927 accession showed no brown discoloration, whereas Atlantic displayed characteristic ZC tuber symptoms (Figure 2C). Furthermore, molecular diagnostic analyses by qPCR and conventional PCR indicated that *Sb*-PI310927 had a very low relative *C*Lso titer of 4.87% compared with Atlantic plants (*C*Lso titer set to 100%) (Figure 2D,E).

### Segregation of ZC Resistance Among True Seed Progeny of *Sb-PI310927*

**Figure 3:**
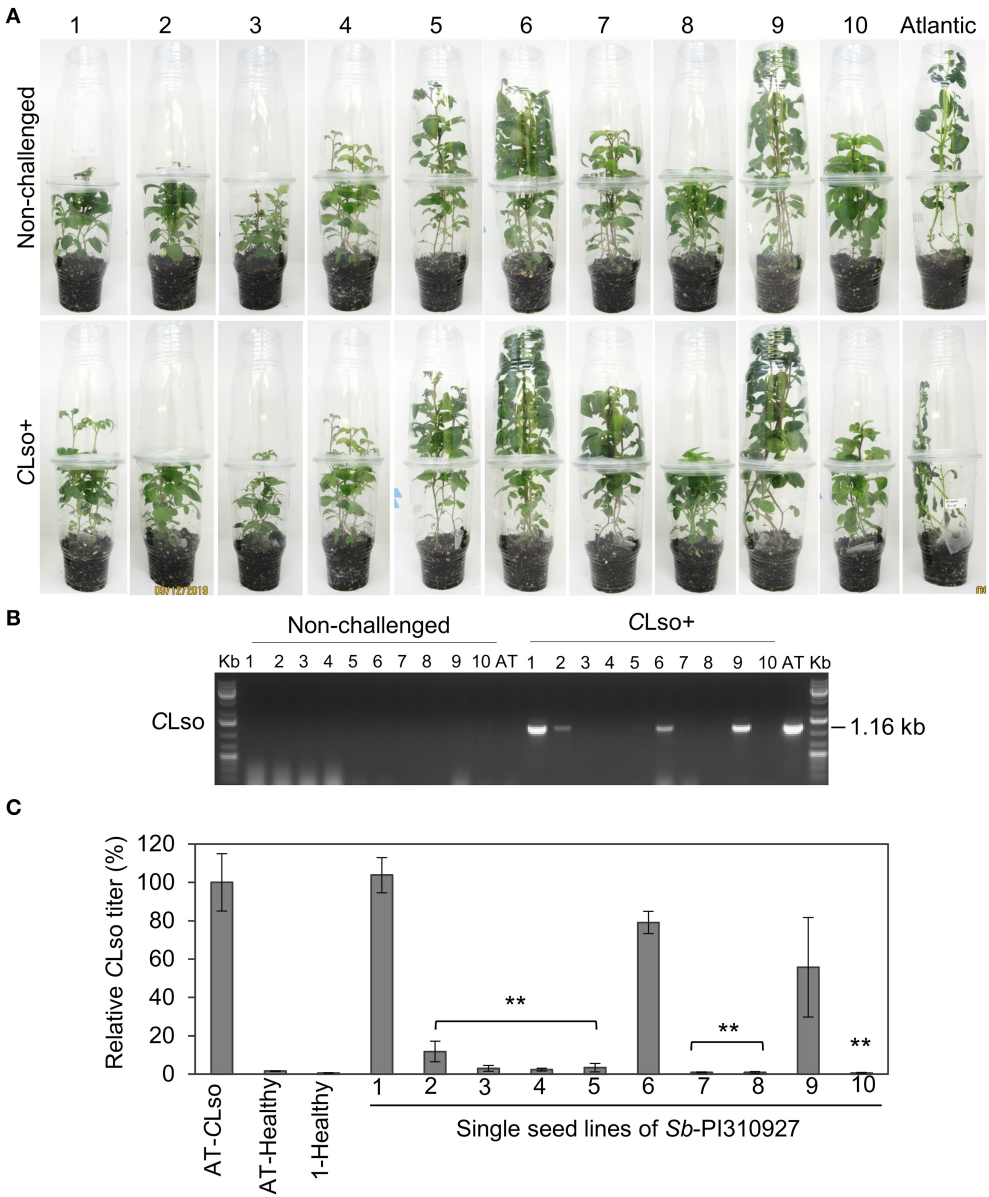
ZC resistance trait evaluation of single seed lines of *Solanum berthaultii* accession PI310927. (**A**) Photos of representative lines 1–10 of *Sb*-PI310927 and Atlantic controls following challenge with *C*Lso^+^ psyllids at 28 dpi. (**B**) Detection of *C*Lso by PCR amplification of *C*Lso 16s rDNA in leaf tissues of 10 single seed lines of *Sb*-PI310927 accession and Atlantic (AT) control plants challenged with *C*Lso^+^ psyllids or no psyllids at 28 dpi. Controls included a positive control, *C*Lso-infected Atlantic, a negative control, healthy Atlantic (H), and water (W). (**C**) Relative quantification of *C*Lso titers of the single seed lines and Atlantic (AT) at 28 dpi by qPCR. Relative *C*Lso titers were calculated from three biological replicates; error bars represent ± standard error of the mean. The *p*-values were calculated by Student's *t*-test relative to the infected control. **P ≤ 0.01.

All screening was conducted using *in vitro* micro-propagated plants from a true seed sourced from the USDA-ARS germplasm bank. As with most wild germplasm collections, *Sb-*PI310927 true seeds could be heterozygous and pooled from multiple plants during germplasm maintenance, thus having the potential for segregating alleles (Bamberg and Del Rio, 2020). To determine if there would be any segregation of the ZC-resistant trait of *Sb-*PI310927 among true seed progeny and to recover a stable resistant line for further studies, 10 true botanical seeds of *Sb-*PI310927 were germinated and multiplied individually by micropropagation. The ZC resistance trait of the 10 single seed lines was then evaluated following no-choice challenges with *C*Lso^+^, *C*Lso^−^, or no psyllids under growth chamber conditions (Figure 3A). At 21 dpi, the susceptible Atlantic control showed characteristic ZC symptoms with high titers of *C*Lso transmission, whereas almost all *Sb-*PI310927 lines did not show any ZC symptoms. However, conventional PCR revealed that only ~50% of *Sb*-PI310927 single seed lines were negative for *C*Lso (Figure 3B). Further confirmation by quantitative PCR showed that six *Sb*-PI310927 single seed lines (#3, 4, 5, 7, 8, and 10) were *C*Lso^−^ or had undetectable levels of *C*Lso. Three lines (#2, 6, and 9) showed low to moderate levels of *C*Lso compared with Atlantic control (*C*Lso titer set to 100%). We also noted some segregation in plant height among the 10 lines, which can be observed even among unchallenged (healthy) plants. Together, these results suggest some degree of segregating alleles among the true seed pools of these wild accessions possible for multiple traits (Bamberg and Del Rio, 2020).

### Host Preference, Fecundity, and Survival of the Psyllids on Sb-PI310927

**Figure 4:**
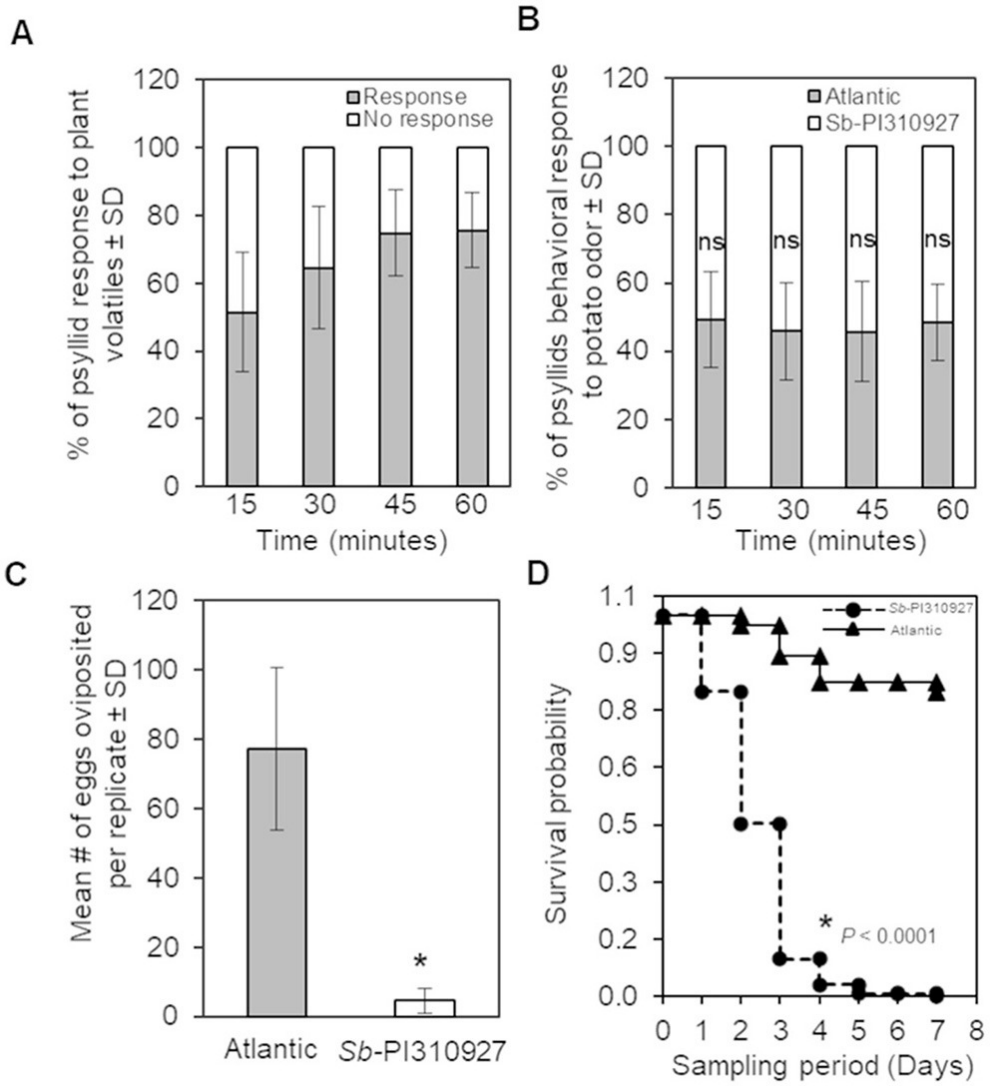
Olfactometer, oviposition, and survival evaluations of *Bactericera cockerelli* adults on *Solanum berthaultii* PI310927. **(A)** Olfactometer (Y-tube) behavioral response of potato psyllid adults to plant volatiles under stable conditions observed every 15 min for a maximum of 60 min. Bar graphs represent the overall mean percentages of adults choosing either odor source ± standard deviation (*n* = 10). **(B)** Potato psyllid's behavioral response to *Sb*-PI310927 and susceptible Atlantic (control). Bar graphs represent the mean percentages of adults ± standard deviation (*n* = 10). **(C)** Female psyllids oviposition at day 7 in no-choice assays using whole plants. Bar graphs represent the mean number of oviposited eggs per replicate ± standard deviation (*n* = 10); the *p*-value was calculated by Student's *t*-test relative to the Atlantic control, ^*^P ≤ 0.0001. **(D)** Survival analysis of potato psyllid adults (*n* = 10) for 7 days showed significant psyllids mortality after exposure to *Sb*-PI310927 when compared with Atlantic plants. The *p-*value was calculated by the Kaplan–Meier analysis, *P* < 0.0001. SD, standard deviation; ns, no significance.

The following two possible mechanisms explain the ZC resistance trait of *Sb*-PI310927: (i) resistance to the psyllid either by antixenosis or antibiosis effects and (ii) immune gene-mediated resistance to the bacteria or the insect. Although not mutually exclusive, we first explored if there were antixenosis or antibiosis effects. For this, we evaluated whether there was a host preference between *Sb*-PI310927 line#10 and Atlantic plants equidistantly placed in an olfactometer-based choice assay (*n* = 10 plants; 20 psyllids/plant). Of the total psyllids analyzed in the experiment (20 psyllids × 10 plants = 200 psyllids), the majority (76%) of them responded within 60 min after the assay was initiated, i.e., reached a 4-cm set threshold distance in the arms toward the plant odor sources (Figure 4A). However, no significant differences in host-selection were found between *Sb-*PI310927 and Atlantic odor sources at the sampling points tested (Figure 4B; 15 min: χ^2^ = 0.053, *df* = 1, *P* = 0.82; 30 min: χ^2^ = 0.644, *df* = 1, *P* = 0.42; 45 min: χ^2^ = 0.65, *df* = 1, *P* = 0.42; and 60 min: χ^2^ = 0.118, *df* = 1, *P* = 0.73).

As there were no differences in host preference, we next evaluated if there were any adverse effects on oviposition, hatching, and survival of psyllids using no-choice assays with 2-month-old *Sb-*PI310927 line#10 and Atlantic plants (*n* = 10 plants; 10 psyllids/plant). The results showed a significant reduction (*F* = 113.68, *df* = 1, *P* < 0.001) in the number of oviposited eggs after 7 days on *Sb-*PI310927 when compared with Atlantic plants (Figure 4C). The few oviposited eggs on *Sb-*PI310927 were also defective in hatching. Furthermore, survival analysis showed that adult psyllids on *Sb-*PI310927 had a significantly (Kaplan Meier analysis, *P* < 0.0001) lowered probability of survival beyond day 3 when compared with Atlantic plants (Figure 4D).

### Leaf Trichome Density and Morphological Differences of *Sb-PI310927*

**Figure 5:**
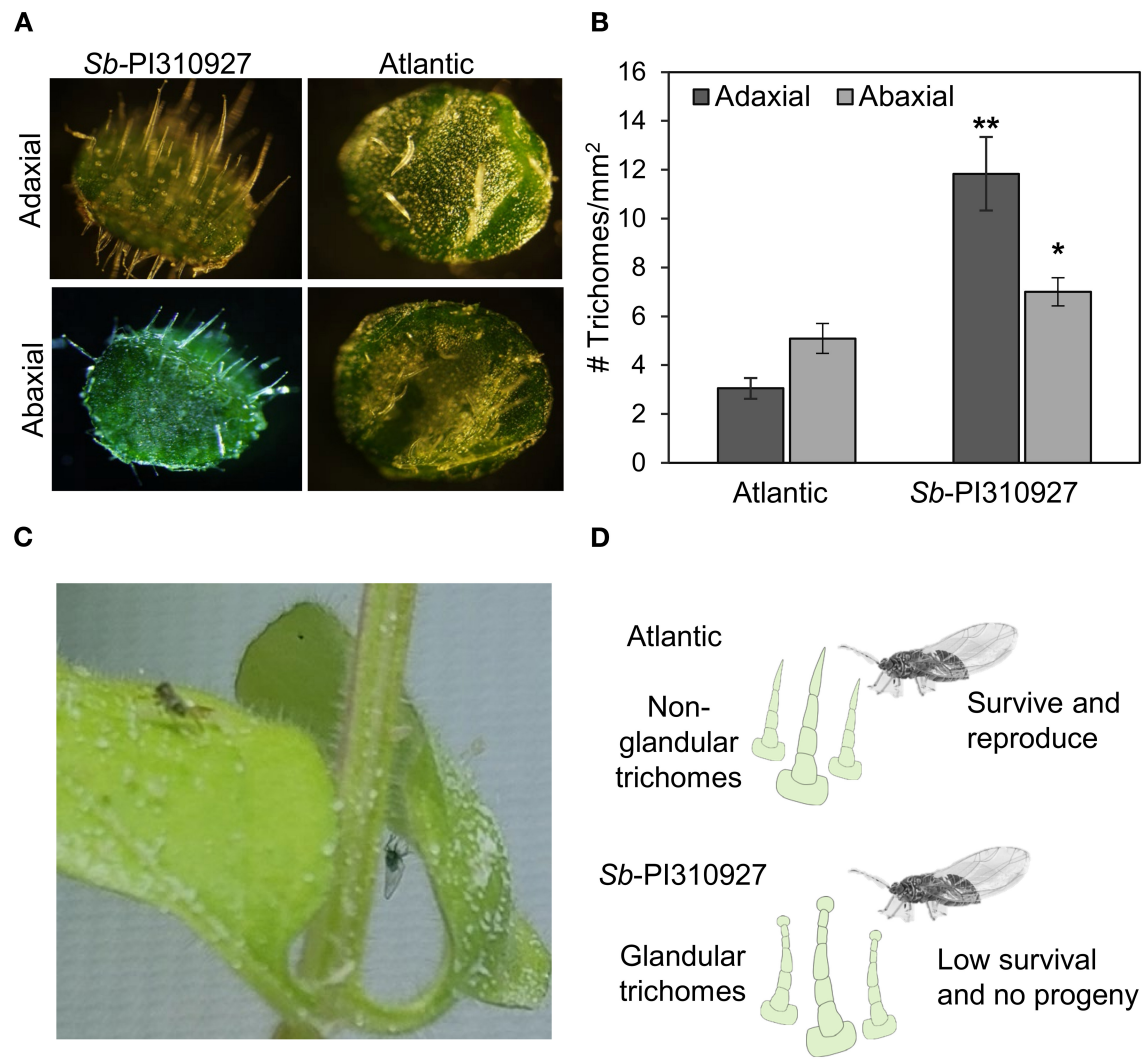
Evaluation of leaf trichomes on *Solanum berthaultii* PI310927 and Atlantic. (**A**) Microscopic evaluation of shape and density of trichomes on the abaxial and adaxial surfaces of leaves of *Sb-*PI310927 line 10 and Atlantic controls. (**B**) Estimation of trichome density on the adaxial and abaxial leaf surfaces of *Sb-*PI310927 and Atlantic controls. Errors bars represent the mean number of trichomes per mm^2^ ± standard error of the mean (*n* = 12). The *p*-values were calculated by Student's *t*-test relative to the Atlantic control. * *P* ≤ 0.01; ** *P* ≤ 0.001. (**C**) Dead psyllids on abaxial and adaxial leaf surfaces of *Sb-*PI310927 plants using non-choice assays. (**D**) Proposed mechanism of negative effects of *Sb-*PI310927 on potato psyllid.

Lastly, in our experiments, we observed that the leaves of *S. berthaultii* were “sticky,” and the movement of psyllids appeared to be impaired (Figure 5C). Hence, under a microscope, we examined the adaxial and abaxial surfaces of 1-month-old Sb-PI310927 line #10. *Sb-*PI310927 leaves had a more significant number of trichomes, and they appear longer than those of Atlantic leaves (Figures 5A,B). The number of trichomes was significantly (*P* < 0.01) higher in *Sb*-PI310927 than in Atlantic leaves on both the adaxial and abaxial surfaces (Figures 5A,B). It is possible that the observed reduced fecundity and survival of psyllids effects on *Sb-*PI310927 could be associated with the increased trichome density and the presence of glandular trichomes (Figures 5C,D).

## Discussion

Zebra chip, a devastating potato disease worldwide, is complex due to its association with the phloem-limited and uncultured *C*Lso and the potato psyllid vector. Using chemical measures has helped control the psyllid population, but it is associated with high costs and positive selection for insecticide resistance (Szczepaniec et al., 2019). In the present study, new sources of ZC resistance were identified among a wild collection of tuber-bearing *Solanum* spp. (*Solanum* sect. *Petota*) (Hardigan et al., 2015). This panel is a taxonomically well-characterized and diverse collection to mine for valuable traits (Hardigan et al., 2015).

Following the screening, phenotypic evaluations, and quantitation of *C*Lso titers of 52 accessions infected with *C*Lso carrying psyllids, we identified one ZC-resistant accession, *S. berthaultii* PI310927, and three ZC moderately tolerant accessions, namely, *Solanum kurtzianum* PI498359, *Solanum okadae* PI498130, and *Solanum raphanifolium* PI310953. Phenotypically, although the three ZC-tolerant wild *Solanum* accessions (*S. kurtzianum* PI498359, *S. okadae* PI498130, and *S. raphanifolium* PI310953) showed some ZC foliar symptoms at 28 dpi, they were able to tolerate and survive *C*Lso. In contrast, the susceptible Atlantic control plants showed severe symptoms, wilting, and eventual death. There are some commonalities among the three tolerant and one resistant accessions. They were all sourced from countries that are relatively closer to the South American continent (Hardigan et al., 2015). The four species are diploids and belong to the same species, clade 4, as described by Spooner and Castillo (1997). Several of these accessions were also previously reported to be tolerant to other potato pests, including green peach aphid, potato aphid, Colorado potato beetle, potato flea beetle, and potato leafhopper (Flanders et al., 1992).

The *S. berthaultii* PI310927 is a highly promising candidate among the ZC-resistant lines. Characterization of resistance among the *S. berthaultii* PI310927 true seeds showed a low-to-moderate level of transmission of *C*Lso among true seed progeny, indicating the presence of heterozygosity or allelic variation among the true seed progeny, which is expected from wild germplasm where traits and alleles are not fixed. Potato breeding efforts with hybrids that incorporated *S. berthaultii* as one of the parents reported variable levels of tolerance to *C*Lso, likely caused due to the heterogeneity of the progeny (Butler et al., 2011; Prager et al., 2013; Rashidi et al., 2017). Our results suggest that natural allelic variations present among the true seed progenies of wild germplasm can impact trait phenotypes and underscore the need to evaluate multiple seed progeny independently propagated.

Importantly, we were able to recover several true seed lines of *S. berthaultii* PI310927 (#3, 4, 5,7, 8, and 10) that showed no ZC symptoms and detectable *C*Lso, when compared with the Atlantic control (100%) (Figures 3B,C). Further characterization of the resistant single seed line #10 showed no differences in psyllid preference to odor sources of *Sb*-PI310927 and Atlantic plants (Figures 4A,B). However, we found greater leaf trichome density that may cause the negative impacts observed on psyllid fecundity and survival (Figures 4C,D, 5A–D). Previous studies on other wild potato relatives and hybrids suggested similar effects of the glandular trichomes against several potato pests (Tingey and Gibson, 1978; Tingey and Laubengayer, 1981; Yencho and Tingey, 1994; Malakar and Tingey, 2000; Butler et al., 2011; Diaz-Montano et al., 2014; Rubio-Covarrubias et al., 2017). Additionally, specific secondary metabolites produced by glandular trichomes were reported to influence host selection (Prager et al., 2018). Further investigation into potential chemical signatures or metabolites synthesized by the *S. berthaultii* PI310927 glandular trichomes may bring new insights into the mechanisms of the observed ZC and psyllid resistance.

## Conclusion

In this study, screening 52 tuber-bearing wild *Solanum* species resulted in identifying a ZC resistant and several tolerant accessions. The resistant *S. berthaultii* accession has dense glandular leaf trichomes. This foliar structural modification could be responsible for much of the observed ZC resistance. We suggest the *S. berthaultii* as a promising source for ZC/psyllid resistance that can be further studied to understand insect resistance mechanisms and incorporated into the potato production system as a trap crop for psyllid or breeding new potato cultivars.

## Data Availability Statement

The original contributions presented in the study are included in the article/Supplementary Material, further inquiries can be directed to the corresponding author/s.

## Author Contributions

KM conceived the project and supervised the study. KM, FI, CA, VA, and MV designed the experiments and interpreted the results. VM, MR, MD, and SI conducted the experiments, analyzed the data, and prepared the manuscript. All authors contributed to the editing and review of the manuscript.

## Funding

This study was supported in part by funds from Texas A&M AgriLife Research Insect-Vectored Disease Seed Grant (124185-96210) and the Texas A&M AgriLife Institute for Advancing Health Through Agriculture, USDA-NIFA (HATCH 1023984) to KM and USDA-NIFA (2019-03814) to MIV. We thank Jesse Schartner and John Bamberg (U.S. Potato Genebank, NRSP-6, Sturgeon Bay, WI) for providing the *Solanum* accessions. We also thank the technical assistance of Denise Rossi, Ashley Jacques, Briana Jacques, and Renesh Bedre (Texas A&M AgriLife Research).

## Conflict of Interest

The authors declare that the research was conducted in the absence of any commercial or financial relationships that could be construed as a potential conflict of interest. The reviewer XW is currently organizing a Research Topic with the author KM.

## Publisher's Note

All claims expressed in this article are solely those of the authors and do not necessarily represent those of their affiliated organizations, or those of the publisher, the editors and the reviewers. Any product that may be evaluated in this article, or claim that may be made by its manufacturer, is not guaranteed or endorsed by the publisher.

## Supplementary Material

Supplementary Figure 1 Morphological diversity among the tuber-bearing *Solanum* section *Petota* species.

Supplementary Figure 2 Phenotypic evaluation of zebra chip (ZC) disease symptoms in wild *Solanum* section *Petota* accessions. Screening of accessions was done using a no-choice feeding assay and *Candidatus* Liberibacter solanacearum-carrying psyllids in the soil in modified cups (**A**) and pots (**B**) inside cages. (**C**) ZC severity index (SI) scale (1, resistant; 2, moderately susceptible; 3, highly susceptible).

Supplementary Figure 3 Preliminary screening of *Solanum* section *Petota* accessions for resistance or tolerance to zebra chip disease, with a no-choice feeding assay with *Candidatus* Liberibacter solanacearum-carrying psyllids. Morphological differences in ZC symptoms of 28-day-old *Solanum* sect. *Petota* accessions.

Supplementary Figure 4 The phenotypes of 4-week-old Atlantic and *Sb*-PI310927 accession zebra chip disease, with a no-choice feeding assay with *Candidatus* Liberibacter solanacearum-carrying psyllids. Morphological differences in ZC symptoms (leaf curling and wilting) of Atlantic (28-day-old) (**A**) and *Sb*-PI310927 (**B**) are shown. A closeup of ZC symptoms is shown in the inset box.

Supplementary Table 1 List of the *Solanum* species and accessions screened for zebra chip resistance.

Supplementary Table 2 List of primers used in this study.
