# Corrigendum: Identification and characterization of potato zebra chip resistance among wild *Solanum* species

**DOI:** 10.3389/fmicb.2025.1605122

**Published:** 2025-04-22

**Authors:** Victoria Mora, Manikandan Ramasamy, Mona B. Damaj, Sonia Irigoyen, Veronica Ancona, Carlos A. Avila, Maria Isabel Vales, Freddy Ibanez, Kranthi K. Mandadi

**Affiliations:** ^1^Texas A&M AgriLife Research and Extension Center, Weslaco, TX, United States; ^2^Department of Agriculture, Agribusiness, and Environmental Sciences, Texas A&M University-Kingsville, Weslaco, TX, United States; ^3^Department of Horticultural Sciences, Texas A&M University, College Station, TX, United States; ^4^Department of Entomology, Texas A&M University, College Station, TX, United States; ^5^Department of Plant Pathology & Microbiology, Texas A&M University, College Station, TX, United States; ^6^Institute for Advancing Health Through Agriculture, Texas A&M AgriLife, College Station, TX, United States

**Keywords:** *Candidatus* Liberibacter solanacearum, Fastidious bacteria, *Bactericera cockerelli*, zebra chip (ZC), wild accessions, resistant traits, antibiosis

In the published article, there was an error in Figure 4 as published. The Figure 4D chart labels corresponding to Atlantic and Sb-PI310927 genotypes were inadvertently reversed during formatting. The corrected Figure 4 and its caption appear below.

**Figure 4:**
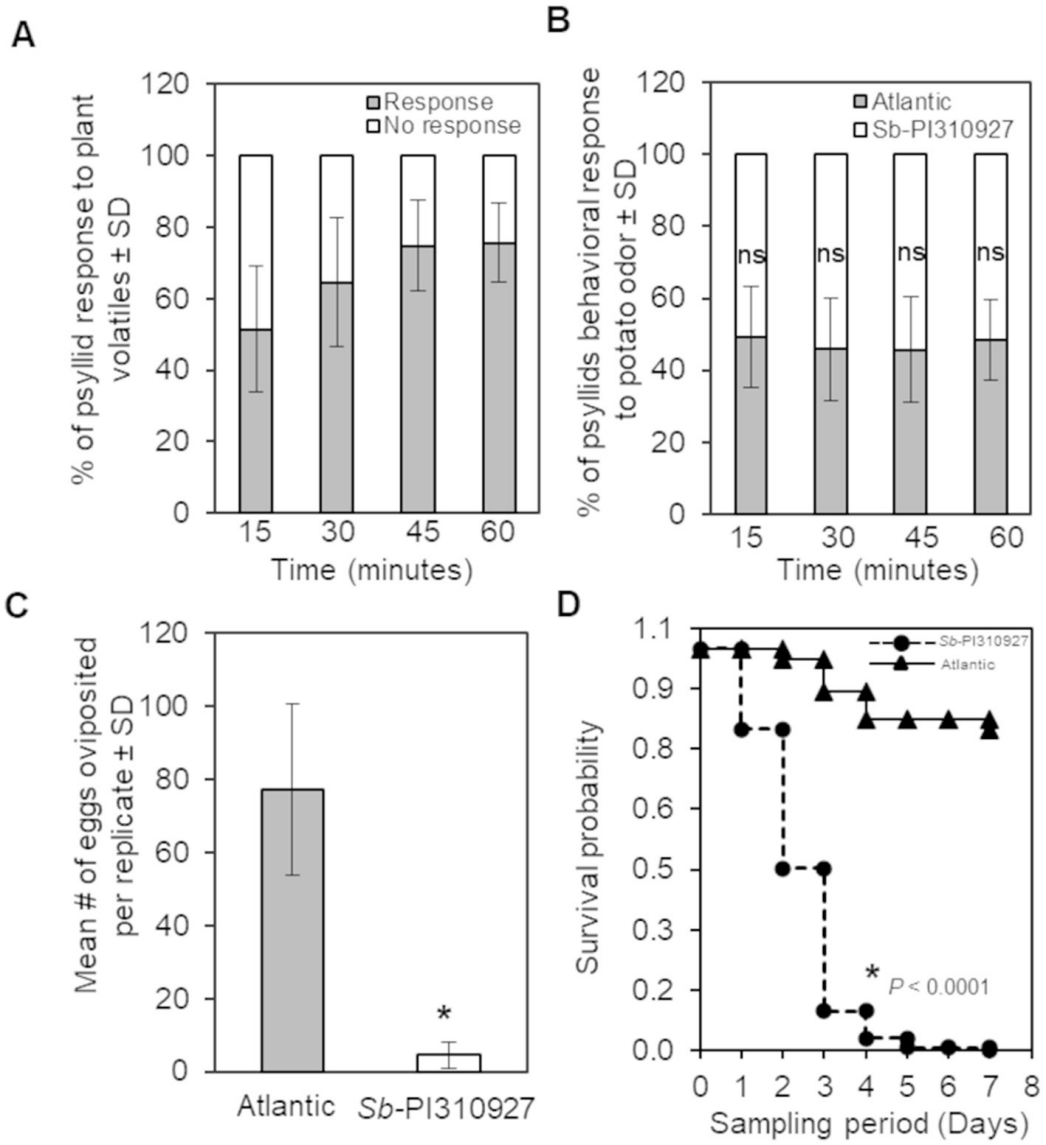
Olfactometer, oviposition, and survival evaluations of *Bactericera cockerelli* adults on *Solanum berthaultii* PI310927. **(A)** Olfactometer (Y-tube) behavioral response of potato psyllid adults to plant volatiles under stable conditions observed every 15 min for a maximum of 60 min. Bar graphs represent the overall mean percentages of adults choosing either odor source ± standard deviation (*n* = 10). **(B)** Potato psyllid's behavioral response to *Sb*-PI310927 and susceptible Atlantic (control). Bar graphs represent the mean percentages of adults ± standard deviation (*n* = 10). **(C)** Female psyllids oviposition at day 7 in no-choice assays using whole plants. Bar graphs represent the mean number of oviposited eggs per replicate ± standard deviation (*n* = 10); the *p*-value was calculated by Student's *t*-test relative to the Atlantic control, ^*^P ≤ 0.0001. **(D)** Survival analysis of potato psyllid adults (*n* = 10) for 7 days showed significant psyllids mortality after exposure to *Sb*-PI310927 when compared with Atlantic plants. The *p-*value was calculated by the Kaplan–Meier analysis, *P* < 0.0001. SD, standard deviation; ns, no significance.

The authors apologize for this error and state that this does not change the scientific conclusions of the article in any way. The original article has been updated.

