# Supplementary material for: Identification and Characterization of Potato Zebra Chip Resistance Among Wild Solanum Species
Source: Front Microbiol. 2022 Jul 27;13:857493. doi: 10.3389/fmicb.2022.857493 (PMC9363700; doi:10.3389/fmicb.2022.857493)
Supplement: Supplementary Figure 1 — Morphological diversity among the tuber-bearing Solanum section Petota species. [file Presentation_1.pdf]

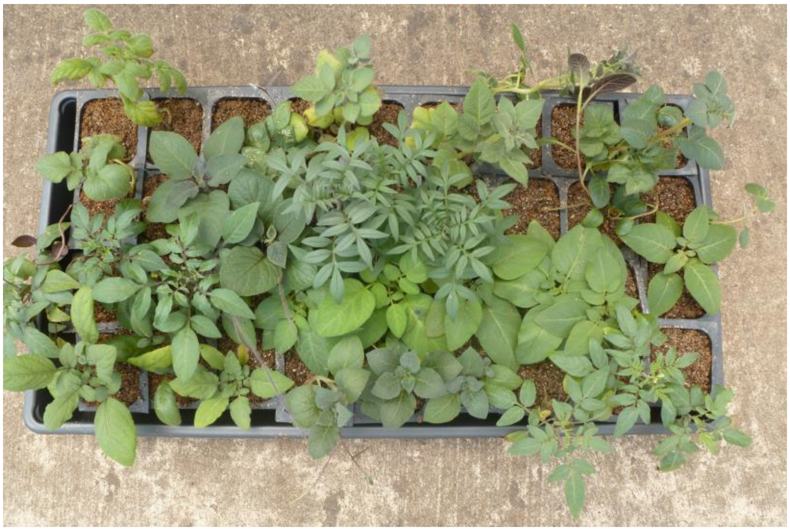

Supplementary Figure 1 Morphological diversity among the tuber-bearing *Solanum* section *Petota* species.

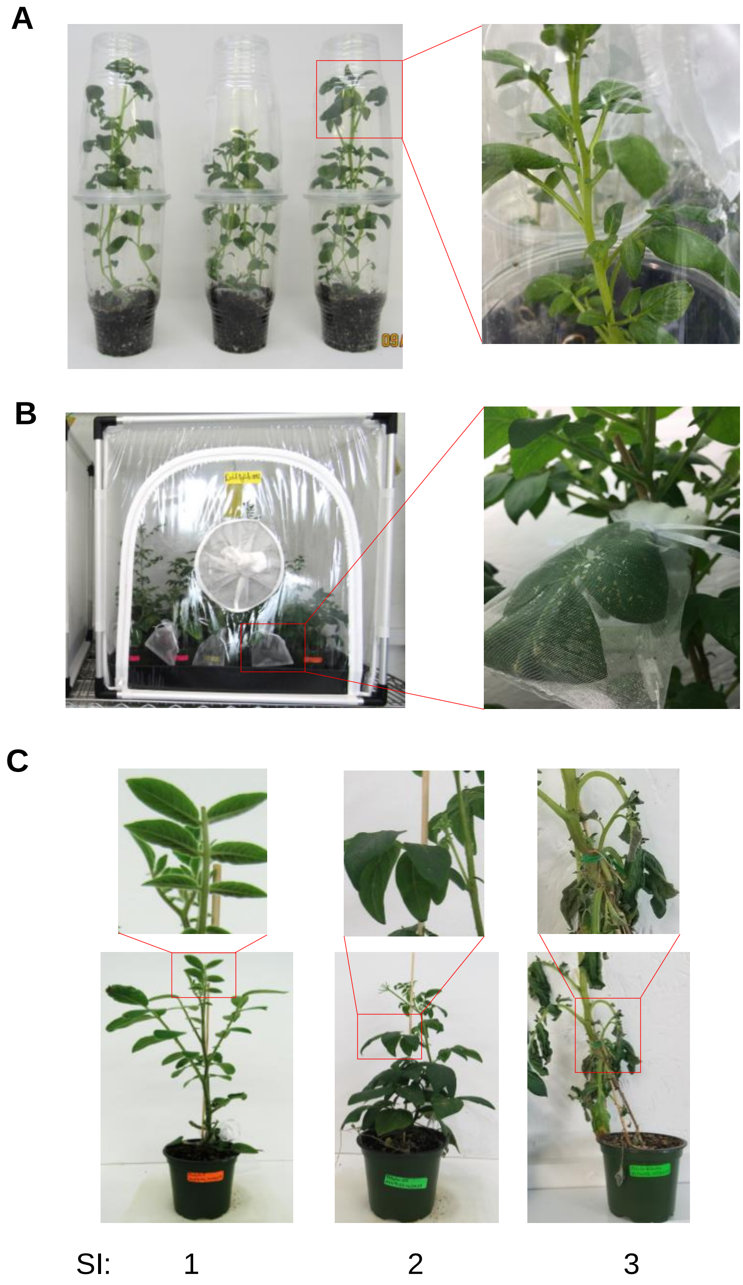

Supplementary Figure 2 Phenotypic evaluation of zebra chip (ZC) disease symptoms in wild *Solanum* section *Petota* accessions. Screening of accessions was done using a no choice feeding assay and *Candidatus* Liberibacter solanacearum-carrying psyllids in soil in modified cups **(A)** and pots **(B)** inside cages. **(C)** ZC severity index (SI) scale (1, resistant; 2, moderately susceptible; 3, highly susceptible).

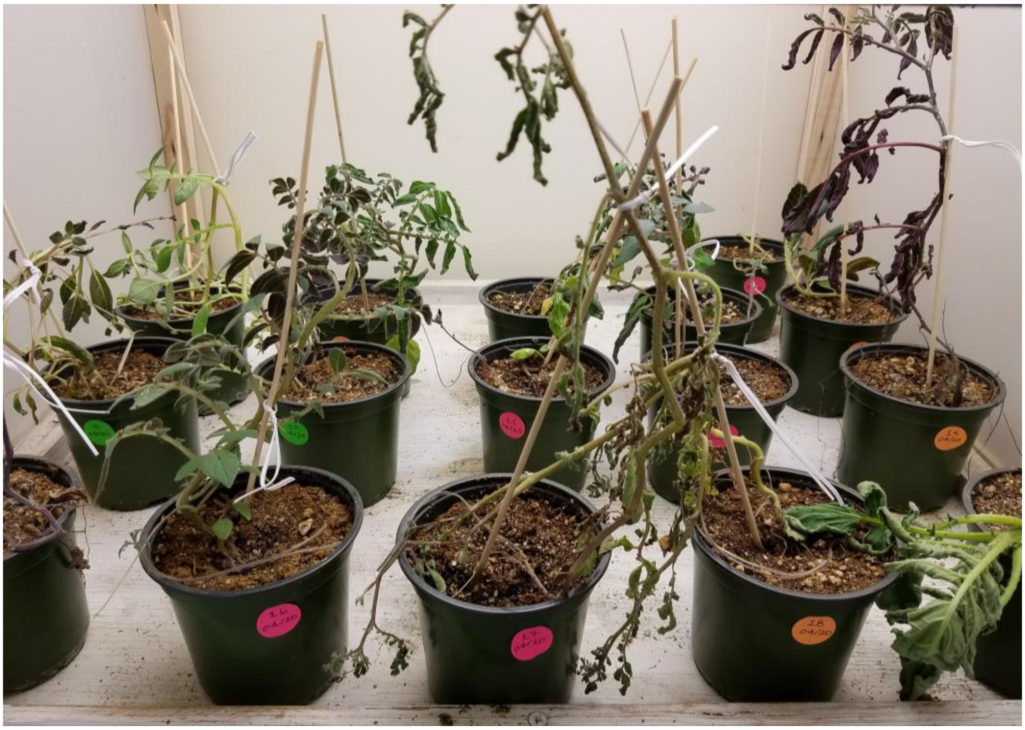

Supplementary Figure 3. Preliminary screening of *Solanum* section *Petota* accessions for resistance or tolerance to zebra chip disease, with a no choice feeding assay with *Candidatus* Liberibacter solanacearum-carrying psyllids. Morphological differences in ZC symptoms of *Solanum* sect. *Petota* accessions (28-day-old)

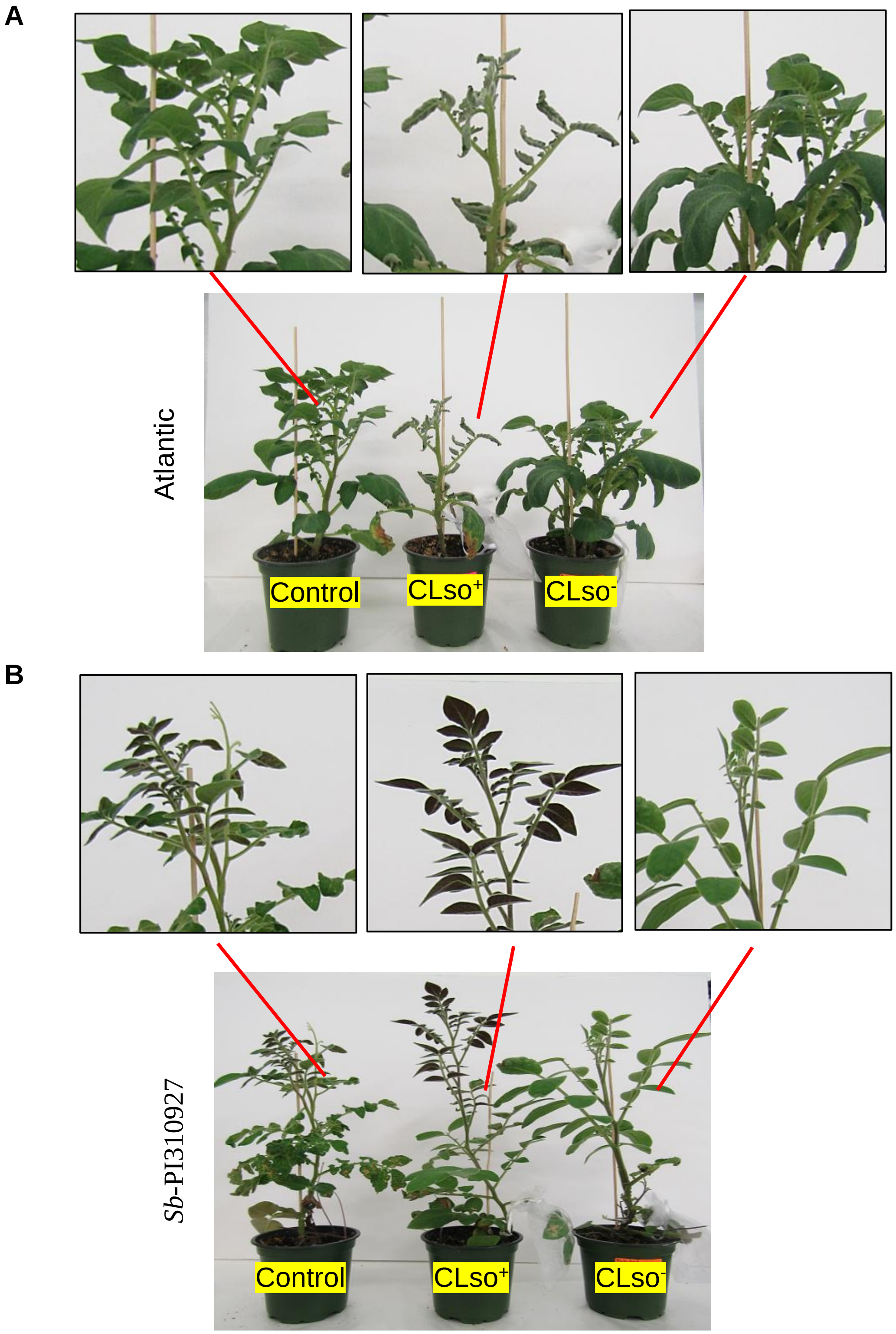

Supplementary Figure 4. The phenotype of four-week-old Atlantic and *Sb*-PI310927 accession zebra chip disease, with a no-choice feeding assay with *Candidatus* Liberibacter solanacearum-carrying psyllids. Morphological differences in ZC symptoms (leaf curling and wilting) of Atlantic (28-day-old) (**A**) and *Sb*-PI310927 (**B**) are shown. A closeup of ZC symptoms is shown in the inset box.
