# Supplementary material for: Identification and Characterization of Potato Zebra Chip Resistance Among Wild Solanum Species
Source: Front Microbiol. 2022 Jul 27;13:857493. doi: 10.3389/fmicb.2022.857493 (PMC9363700; doi:10.3389/fmicb.2022.857493)
Supplement: Supplementary Table 1 — List of the Solanum species and accessions screened for zebra chip resistance. [file Data_Sheet_1.docx]

**Supplementary Table 1.** List of the *Solanum* species and accessions screened for zebra chip resistance.

| ***Solanum* species** | **Accession** | **Ploidy** | **Clade^*^** | **Series^§^** |
| --- | --- | --- | --- | --- |
| *S. acaule* | PI175395 | 4x | 4 | Acaulia |
| *S. acaule* | PI472661 | 4x | 4 | Acaulia |
| *S. acaule* | PI473481 | 4x | 4 | Acaulia |
| *S. berthaultii* | PI458365 | 2x | 4 | Tuberosa |
| *S. berthaultii* | PI498141 | 2x | 4 | Tuberosa |
| *S. berthaultii* | PI310927 | 2x | 4 | Tuberosa |
| *S. boliviense* | PI265873 | 2x | 4 | Megistacroloba |
| *S. boliviense* | PI545964 | 2x | 4 | Megistacroloba |
| *S. boliviense* | PI597736 | 2x | 4 | Megistacroloba |
| *S. brevicaule* | PI265579 | 4x | 4 | Tuberosa |
| *S. brevicaule* | PI473011 | 2x | 4 | Tuberosa |
| *S. brevicaule* | PI473062 | 2x | 4 | Tuberosa |
| *S. brevicaule* | PI435079 | 2x | 4 | Tuberosa |
| *S. brevicaule* | PI473185 | 6x | 4 | Tuberosa |
| *S. brevicaule* | PI473190 | 6x | 4 | Tuberosa |
| *S. brevicaule* | PI205407 | 2x | 4 | Tuberosa |
| *S. brevicaule* | PI472978 | 2x | 4 | Tuberosa |
| *S. brevicaule* | PI500053 | 2x | 4 | Tuberosa |
| *S. bulbocastanum* | PI545751 | 2x | 2 | Bulbocastana |
| *S. bulbocastanum* | PI243510 | 2x | 2 | Bulbocastana |
| *S. bulbocastanum* | PI275188 | 2x | 2 | Bulbocastana |
| *S. candolleanum* | PI265863 | 2x | 4 | Tuberosa |
| *S. candolleanum* | PI365321 | 2x | 4 | Tuberosa |
| *S. candolleanum* | PI458379 | 2x | 4 | Tuberosa |
| *S. chacoense* | PI197760 | 2x | 4 | Yungasensa |
| *S. chacoense* | PI275139 | 2x | 4 | Yungasensa |
| *S. chacoense* | PI320293 | 2x | 4 | Yungasensa |
| *S. circaeifolium* | PI498116 | 2x | 4 | Circaeifolia |
| *S. circaeifolium* | PI498120 | 2x | 4 | Circaeifolia |
| *S. commersonii* | PI472837 | 2x | 4 | Commersoniana |
| *S. commersonii* | PI473411 | 2x | 4 | Commersoniana |
| *S. commersonii* | PI558050 | 2x | 4 | Commersoniana |
| *S. demissum* | PI160208 | 6x | 4 | Demissa |
| *S. demissum* | PI230589 | 6x | 4 | Demissa |
| *S. demissum* | PI498232 | 6x | 4 | Demissa |
| *S. hjertingii* | PI251065 | 4x | 4 | Longipedicellata |
| *S. hjertingii* | PI283103 | 4x | 4 | Longipedicellata |
| *S. hjertingii* | PI545715 | 4x | 4 | Longipedicellata |
| *S. infundibuliforme* | PI265867 | 2x | 4 | Cuneolata |
| *S. infundibuliforme* | PI458324 | 2x | 4 | Cuneolata |
| *S. infundibuliforme* | PI472894 | 2x | 4 | Cuneolata |
| *S. jamesii* | PI458425 | 2x | 1 | Pinnatisecta |
| *S. jamesii* | PI592422 | 2x | 1 | Pinnatisecta |
| *S. jamesii* | PI605370 | 2x | 1 | Pinnatisecta |
| *S. kurtzianum* | PI498359 | 2x | 4 | Tuberosa |
| *S. microdontum* | PI458355 | 2x | 4 | Tuberosa |
| *S. okadae* | PI498130 | 2x | 4 | Tuberosa |
| *S. pinnatisectum* | PI347766 | 2x | 1 | Pinnatisecta |
| *S. raphanifolium* | PI310953 | 2x | 4 | Megistacroloba |
| *S. stoloniferum* | PI161170 | 4x | 4 | Longipedicellata |
| *S. tuberosum subsp. andigena* | PI320377 | 2x | 4 | Tuberosa |
| *S. verrucosum* | PI498062 | 2x | 4 | Tuberosa |

^*^Potato species taxonomic clade described by Spooner and Castillo (1997).

**^§^**Potato species taxonomic series described by Hawkes (1990).

**Supplementary Table 2.** List of primers used in this study.

| Primer | Sequence (5´-3´) | Annealing temperature | Product size |
| --- | --- | --- | --- |
| Conventional PCR |  |  |  |
| RPL2-F  RPL2-R | GAG GGC GTA CTG AGA AAC CA CTT TTG TCC AGG AGG TGC AT | 50ºC | 0.18 kb |
| OI2C-F  OA2-R | GCC TCG CGA CTT CGC AAC CCA T  GCG CTT ATT TTT AAT AGG AGC GGC A | 68ºC | 1.16 kb |
| Quantitative PCR |  |  |  |
| Sotu-RPL2-F  Sotu-RPL2-R | GTG GAG GAC GAA CTG AGA AA  AGT CCT CCT TGC AGC AAT AA | 55ºC | 0.17 kb |
| Lso-F  HLB-R | CGA GCG CTT ATT TTT AAT AGG AGC  GCG TTA TCC CGT AGA AAA AGG TAG | 55ºC | 0.78 kb |

Kb: kilobase pair
